# Comparison of aerobic and combined aerobic and resistance training on low-density lipoprotein cholesterol concentrations in men

**Published:** 2009-10

**Authors:** Ina Shaw, Brandon S Shaw, Oleksandr Krasilshchikov

**Affiliations:** Department of Marketing and Sport Management, Vaal University of Technology, Vanderbijlpark; Department of Sport, Rehabilitation and Dental Sciences, Tshwane University of Technology, Pretoria; Exercise and Sports Science Programme, School of Health Sciences, Universiti Sains, Malaysia

## Abstract

**Summary:**

While aerobic training and, to a lesser degree, resistance training are known to reduce blood concentrations of low-density lipoprotein cholesterol (LDL-C), little is known about the effects of a combination of aerobic and resistance training on LDL-C concentrations. The aim of the investigation was to examine the effects of 16 weeks of no exercise, aerobic training or a combination of aerobic and resistance training on lowering blood concentrations of LDL-C.

Thirty-eight healthy, previously untrained men (mean age: 25 years and six months) with borderline high blood LDL-C concentrations volunteered to participate in this investigation. Each subject’s blood LDL-C concentrations were measured following a nine- to 12-hour fasting period and prior to any exercise. Aerobic training consisted of exercise using a combination of treadmills, rowers, steppers and cycle ergometers. Combined aerobic and resistance training consisted of a combination of aerobic training at 60% of heart rate maximum, and resistance training using eight prescribed exercises performed for two sets of 15 repetitions at 60% of the estimated one-repetition maximum (1-RM).

The no-exercise group was found to have had no significant (*p* ≤ 0.05) change in blood LDL-C concentrations (from 4.12 ± 0.27 to 4.21 ± 0.42 mmol.l^-1^), whereas the aerobic training and combined training groups showed significant and similar (*p* = 0.123) decreases in blood LDL-C concentrations (from 3.64 ± 2.87 to 2.87 ± 0.64 mmol.l^-1^ and from 4.39 ± 1.04 to 3.23 ± 0.71 mmol.l^-1^, respectively). This investigation indicates that a larger dose of aerobic exercise does not necessarily equate to a greater improvement in LDL-C concentrations if the lost aerobic exercise time is replaced with resistance exercise.

## Summary

It has long been known that exercise training can reduce the incidence of cardiovascular disease (CVD) by modifying lipoprotein and lipid concentrations.^1^,^2^ Specifically, exercise has been shown to reduce the main plaque- and thrombosis-forming lipoprotein cholesterol, namely low-density lipoprotein cholesterol (LDL-C).^3^ Exercise can lower LDL-C concentrations since it results in increased lipoprotein and lipid metabolism, presumably via an increased activity of cholesterol ester transfer protein (CETP), lecithin-cholesterol acyl transferase (LCAT) and lipoprotein lipase (LPL). This increased enzymatic activity then increases the muscle fibre’s ability to oxidise fatty acids originating from plasma or very low-density lipoprotein (VLDL) triglycerides.^4^,^5^

Exercise studies have primarily focused on the effects of aerobic exercise and it is regarded as one of the fundamental ways to alter unfavourable LDL-C concentrations in untrained men.^6^-^12^ However, there are significant questions regarding the effects of type, duration and frequency of training required for changes in lipoprotein lipids.^8^ In this regard, it is unclear whether resistance training or the addition of resistance training to aerobic training is as effective at lowering blood LDL-C concentrations as aerobic training.^8^,^13^ Although a plethora of research exists, results from resistance training studies are conflicting,^8^ with some researchers indicating that resistance training lowers blood LDL-C concentrations,^13^-^21^ and others showing that it results in no substantive changes in blood LDL-C concentrations.^5^,^22^-^25^

While evidence exists that aerobic training and possibly resistance training can lower blood LDL-C concentrations, research on the effect of combined aerobic and resistance training on lipoprotein lipids has been sparse and has shown inconsistent results.^14^,^26^,^27^ This is disconcerting since the American College of Sports Medicine recommends a combination of aerobic and resistance training to improve health components.^4^ Similarly, Pierson *et al.*^28^ states that the two major goals of cardiac rehabilitation are to improve physical functioning and reduce CVD risk factors. Therefore, a finding that a combination of aerobic and resistance training can lower LDL-C concentrations would be of critical importance due to the simultaneous synergistic benefits to be gained from each mode of exercise.^29^

Furthermore, although aerobic exercise may result in favourable blood LDL-C concentrations, it also results in loss of protein or muscle mass, effectively lowering resting metabolic rate and fat metabolism.^4^,^18^,^25^ Therefore, by combining aerobic and resistance training, excess protein loss may be overcome to maintain muscle mass and produce favourable increases in resting metabolic rate and fat metabolism. This may possibly result in an increased stimulus for lowering blood LDL-C concentrations over that of aerobic training alone.^14^

Accordingly, the purpose of the investigation was to determine the effects of 16 weeks of aerobic training (AT) and a combination of aerobic and resistance training (CT) on blood LDL-C concentrations. The aim was to compare the effectiveness of these modes of exercise training on lowering blood LDL-C concentrations and to determine if the addition of resistance training to aerobic training during a workout creates an additional impetus when attempting to lower blood LDL-C concentrations. It was hypothesised that the CT would result in similar or greater decreases in blood LDL-C concentrations when compared to AT, despite the subjects in the CT group doing only half as much aerobic training as the aerobic-only training group.

## Methods

Thirty-eight male volunteers with borderline high blood LDL-C concentrations^4^,^30^ were matched by age and assigned to a non-exercising control group (NE) (*n* = 12), an aerobic training (AT) group (*n* = 12), or a combination training (CT) group that used both aerobic and resistance training (*n* = 13). The characteristics of the subjects are shown in Table 1.

**Table 1:** Subject Baseline Descriptive Data

*Variables*	*Non-exercising control group (n = 12)*	*Aerobic training group (n = 13)*	*Combination training group (n = 13)*
Age (years)	25 ± 2.4	25 ± 5.6	26 ± 3.1
Height (cm)	179.3 ± 11.9	176.8 ± 3.8	178.7 ± 7.0
Body mass (kg)	80.3 ± 12.8	74.7 ± 8.2	85.0 ± 12.8
Body fat (%)	17.86 ± 7.86	15.57 ± 6.65	22.04 ± 11.39
Chest press 1-RM (kg)	48.33 ± 14.83	47.00 ± 4.67	56.23 ± 10.23
VO_2max_ (ml.kg^-1^.min^-1^)	27.41 ± 3.59	35.71 ± 4.39	26.81 ± 6.62

Values are means ± standard deviation.

All subjects provided written informed consent following approval of the Institutional Review Board at the Rand Afrikaans University. Subjects were free to discontinue the study at any time. All subjects were male between the ages of 20 and 35 years, were weight stable and had had no previous participation in any regular exercise and dietary programme at least six months prior to the investigation,^13^,^20^,^21^,^31^ were not using any androgens or other drugs known to affect blood LDL-C concentrations^17^ and were free of medical conditions prohibiting exercise.^13^,^28^ All tests took place in the post-absorptive state before a training session and 48 hours after the last training session, to standardise the timecourse changes in blood LDL-C concentrations.^32^

## Body composition measurement

Body mass was measured to the nearest 0.1 kg on a calibrated medical scale (Mettler DT Digitol, Mettler-Toledo AG, Ch-8606 GreiFensee, Switzerland) with the subjects wearing only running shorts. Body fat percentage was determined using a manual skinfold calliper (Harpenden John Bull, British Indicators Ltd., England) and calculated according to the seven-skinfold method of Jackson and Pollock.^33^

## LDL-C analysis

At the start and completion of the experimental period (weeks 0 and 16), following a nine- to 12-hour fasting period and prior to any exercise, blood samples were drawn using the fingerprick method. Samples were drawn while the subject was in the seated position and analysed immediately for LDL-C concentrations as per the Reflotron® system (Roche Products Pty Ltd, Randburg, South Africa) requirements.

The Reflotron® system utilises a within-series precision with a standard deviation of < 0.2% reflectance. The electronics of the Reflotron® system ensured, by a compensation procedure, that mains-frequency synchronous or persistent interference such as extraneous light or zero currents and ultrasound or diathermy equipment (at least 2 m away) did not influence the tests. A measurement accuracy of ± 0.5% reflectance was attained with the Reflotron® system. A reference procedure employed by the Reflotron® system eliminated errors caused by differing component tolerances or aging. The Reflotron® system uses strictly selected light-emitting diodes with wavelengths centred on 567, 642 and 951 nm serving as light sources. The Reflotron® system controlled all functions of the test such as the performance of the test, temperature control, automatic calibration and evaluation of the reflectance measurements, including calculation of the result of a fully automated microprocessor-controlled reflectance photometer.

## Dietary assessment

Subjects were required to complete baseline and post-training seven-day dietary recall, specifying the type and quantity of food and fluids. Portion sizes were illustrated with the aid of measuring cups, glasses, bowls and food items and the recalls were analysed for the daily intake of saturated, mono-unsaturated and polyunsaturated fats, cholesterol and fibre, using the Dietary Manager® computer-based software program (Dietary Manager, Program Management, South Africa).

## Strength measurement

Due to the inactive nature of the subjects, all underwent a preliminary 10-repetition maximum (10-RM) strength test on each of the prescribed resistance training exercises to establish the one-repetition maximum (1-RM).^34^ This test began with a five-minute warm-up and stretching and was followed by five to 10 repetitions of each of the prescribed exercises at 40 to 60% of their estimated 1-RM. Following this, each subject rested and stretched the muscle/muscle group concerned and then performed 10 repetitions at approximately 70% of their estimated 1-RM. If the subject was successful at performing 10 repetitions, the weight was increased conservatively and the subject rested for three to five minutes before attempting to complete another 10 repetitions at the new weight increment.

This protocol was continued until each subject completed no more than 10 repetitions. The maximum weight that could be lifted for 10 repetitions was used to determine 1-RM from the following formula:^34^

$1-RM=\;\frac{weight\;lifted}{1.0278-(reptitions\;to\;fatigue\;\times 0.0278)}$

To determine the number of repetitions to be used for crunches (modified sit-ups) in the training sessions, each subject had to perform as many crunches as possible in one minute.^21^

## Maximal oxygen consumption measurement

Maximal oxygen consumption (VO_2max_) was measured directly at the start and completion of the experimental period using a continuous on-line oxygen analyser (OXYCON-5, Mijnhardt, Bunnik, Netherlands) and cycle ergometer (Monark Ergo Trainer 810, Monark, Vargberg, Sweden). Readings of oxygen consumption (VO_2_) were recorded every 30 seconds over the test period using the metabolic cart (OXYCON-5, Mijnhardt, Bunnik, Netherlands) and a Hans Rudolph two-way non-rebreathing valve (T-shape configuration, model 2700B) (Wynadotte, Kansas City, USA). VO_2max_ was determined at an altitude of 1 650 m above sea level and at a controlled temperature of 21 to 23°C. The graded YMCA cycle ergometry protocol was utilised and if needed, extra stages of 25 W were added to ensure that all subjects reached their respective maximums.

The VO_2max_ test was terminated when any one of the following criteria was met: VO_2_ or heart rate decreased or remained unchanged in response to increases in workload, a respiratory quotient value of 1.14 was reached, a rating of perceived exertion of 20 was reached, the subjects experienced severe leg muscle fatigue or requested to stop.^34^ Workload was also considered maximal when successive increments in work intensity resulted in VO_2_ differences of not more than 1 ml of oxygen per kg per minute.

## Training design

The subjects in the exercise training groups trained three times a week for 16 weeks. Each exercise session began with five minutes of cycling^13^,^18^ and stretching, while five minutes of cycling was used as a cool-down following each session.^13^,^18^

The AT subjects were required to exercise for 45 minutes at 60% of their individual age-predicted maximum heart rates^14^,^28^ using treadmills, rowers, steppers and cycle ergometers.^28^,^31^,^35^ Age-predicted maximum heart rates were determined by subtracting their age from 220. Intensity was increased by 5% every four weeks^14^,^28^ (65, 70 and then 75% of their individual age-predicted maximum heart rate) by increasing treadmill, rower, stepper and/or cycle ergometer speed and/or resistance (grade or tension).

To equalise for duration across the three exercise groups, the CT protocol used resistance training and aerobic training of equal durations. The aerobic component required the CT subjects to exercise at 60% of their individual age-predicted heart rate maximum for 22 minutes, using a combination of treadmills, rowers, steppers and cycle ergometers. The resistance training component consisted of eight resistance training exercises performed for two sets^21^ of 15 repetitions^28^ at 60% of their 1-RM,^13^,^21^ using Polaris® resistance training machines and York® free weights. The prescribed exercises included shoulder press, latissimus dorsi pull-downs, seated chest press, low-pulley row, crunches, unilateral leg press, unilateral knee extensions and unilateral prone hamstring curls. While the aerobic training intensity was similarly adjusted to that of the AT protocol, the 10-RM strength testing protocol took place every four weeks and the newly calculated 1-RM was utilised to adjust resistance training intensity.^13^,^21^

The NE subjects were instructed to maintain their usual activities and not to take part in any form of structured exercise during the 16-week period.

## Statistics

Statistical analysis consisted of basic statistics to determine baseline and post-training means and standard deviations. A pairedsamples *t*-test was applied to determine if a significant change took place in LDL-C at post-training. Differences in LDL-C were compared using a one-way analysis of variance (ANOVA) and a Dunnett T3 *post-hoc* analysis. Spearman’s *rho* was utilised to measure the strength of the linear relationship between LDL-C and the measured extraneous variables. A probability value of ≤ 0.05 was considered significant. Data were analysed using the Statistical Package of Social Sciences (SPSS) version 14 (Chicago, IL).

## Results

**Fig. 1.**
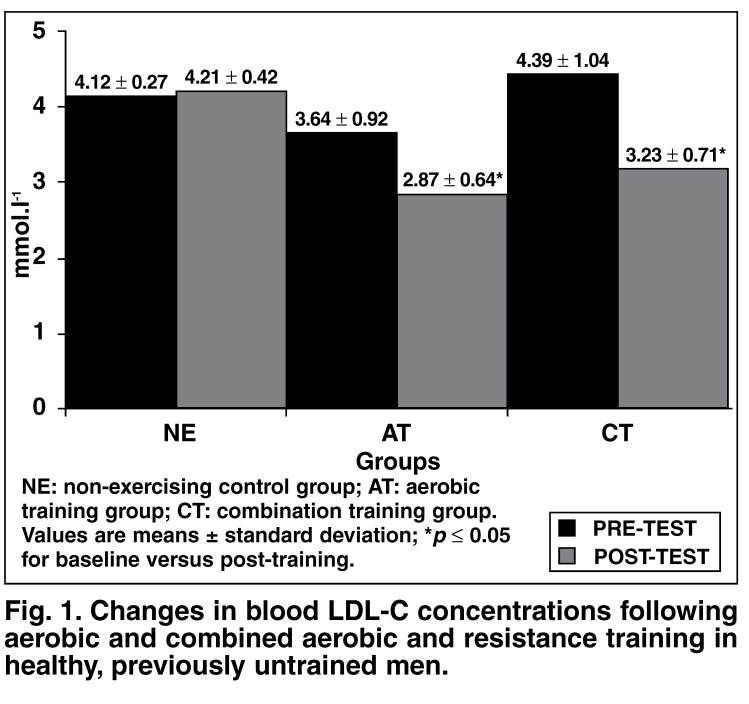
Changes in blood LDL-C concentrations following aerobic and combined aerobic and resistance training in healthy, previously untrained men.

Sixteen weeks of no exercise did not result in a significant (*p* ≤ 0.05) change in blood LDL-C concentrations (Fig. 1). However, the AT programme resulted in a significant mean 0.78 ± 0.65 mmol.l-1 decrease in blood LDL-C concentrations, and the CT programme in a significant 1.16 ± 0.73 mmol.l-1 decrease in blood LDL-C concentrations. Further *post-hoc* analysis revealed no difference in the effectiveness of AT and CT in reducing blood LDL-C concentrations (*p* = 0.123).

The NE and CT group subjects did not demonstrate a significant change in body mass (*p* = 0.398 and *p* = 0.957, respectively), whereas the AT group participants’ body mass decreased significantly (*p* = 0.004) (Table 2). While the NE group was found to have no change in percentage body fat (*p* = 1.000), both the AT and CT groups demonstrated significant decreases in percentage body fat (*p* = 0.002 and *p* = 0.002, respectively).

**Table 2:** Changes In Physical And Dietary Variables Following Aerobic And Combined Aerobic And Resistance Training In Healthy, Previously Untrained Men

*Parameter*	*Non-exercising control group (n = 12)*	*Aerobic training group (n = 13)*	*Combination training group (n = 13)*
Body mass (kg): baseline	80.31 ± 12.84	74.72 ± 8.21	85.00 ± 12.78
Body mass (kg): post-training	79.59 ± 10.93	72.32 ± 7.35*	85.06 ± 10.97
Body fat (%): baseline	17.86 ± 7.86	15.57 ± 6.65	22.04 ± 11.39
Body fat (%): post-training	17.79 ± 8.03	11.89 ± 4.64*	16.95 ± 8.98*
Fat intake (g): baseline	100.31 ± 28.43	91.04 ± 42.09	91.92 ± 27.62
Fat intake (g): post-training	99.20 ± 30.80	77.05 ± 62.10*	67.93 ± 26.18*
Saturated fat intake (g): baseline	35.30 ± 10.91	34.17 ± 16.76	35.41 ± 11.32
Saturated fat intake (g): post-training	37.48 ± 10.48	27.99 ± 19.52	23.63 ± 11.11*
Mono-unsaturated fat intake (g): baseline	37.95 ± 12.75	34.89 ± 18.28	33.79 ± 13.73
Mono-unsaturated fat intake (g): post-training	34.55 ± 13.77	25.98 ± 17.68*	26.08 ± 10.64*
Polyunsaturated fat intake (g): baseline	15.42 ± 9.61	13.20 ± 6.63	11.82 ± 5.03
Polyunsaturated fat intake (g): post-training	16.24 ± 10.38	14.68 ± 24.10	11.70 ± 5.74
Cholesterol intake (g): baseline	360.63 ± 165.86	383.01 ± 213.14	361.97 ± 208.11
Cholesterol intake (g): post-training	389.74 ± 217.82	251.50 ± 141.40*	238.90 ± 81.65*
Fibre intake (g): baseline	19.53 ± 7.76	19.27 ± 8.43	17.74 ± 12.84
Fibre intake (g): post-training	16.53 ± 5.25	16.75 ± 8.99	14.91 ± 12.56
Shoulder press strength (kg): baseline	41.50 ± 14.22	43.83 ± 5.42	53.85 ± 11.70
Shoulder press strength (kg): post-training	73.83 ± 15.24	52.00 ± 10.05*	109.54 ± 15.01*
Lattisimus dorsi pull-down strength (kg): baseline	53.25 ± 13.60	50.33 ± 3.11	57.46 ± 9.73
Lattisimus dorsi pull-down strength (kg): post-training	53.08 ± 15.23	60.67 ± 7.90*	88.92 ± 6.28*
Seated chest press strength (kg): baseline	48.33 ± 14.83	47.00 ± 4.67	56.23 ± 10.23
Seated chest press strength (kg): post-training	46.67 ± 12.91	57.58 ± 6.52*	86.15 ± 11.43*
Low-pulley row strength (kg): baseline	46.33 ± 9.06	47.67 ± 4.12	54.77 ± 6.03
Low-pulley row strength (kg): post-training	43.92 ± 9.17	59.50 ± 8.08*	92.23 ± 7.70*
Crunches strength (reps.min^-1^): baseline	43.42 ± 12.69	42.67 ± 5.63	62.46 ± 15.78
Crunches strength (reps.min^-1^): post-training	42.50 ± 12.51	68.08 ± 14.49*	105.92 ± 14.49*
Leg press strength (kg): baseline	80.67 ± 16.33	87.08 ± 12.67	100.92 ± 13.62
Leg press strength (kg): post-training	78.75 ± 19.46	108.42 ± 10.51*	149.69 ± 19.01*
Knee extension strength (kg): baseline	18.00 ± 2.34	20.50 ± 3.09	23.69 ± 2.95
Knee extension strength (kg): post-training	19.00 ± 2.95	30.83 ± 3.16*	45.00 ± 5.94*
Hamstring curl strength (kg): baseline	10.17 ± 2.72	14.83 ± 2.72	16.54 ± 4.48
Hamstring curl strength (kg): post-training	13.33 ± 3.45	22.25 ± 4.14*	30.23 ± 2.77*
VO_2max_ (ml.kg^-1^.min^-1^): baseline	27.41 ± 3.59	35.71 ± 4.39	26.81 ± 6.62
VO_2max_ (ml.kg^-1^.min^-1^): post-training	26.49 ± 4.55	40.39 ± 6.27*	34.74 ± 5.92*

Values are means ± standard deviation; *p ≤ 0.05 compared to pre-test.

Significant changes were found in the total fat intake following AT and CT participants (*p* = 0.041 and *p* = 0.028, respectively) but not in the NE group (*p* = 0.937) (Table 2). While the CT group significantly decreased their daily intake of saturated fat (*p* = 0.023), the AT and NE group’s intake remained unchanged (*p* = 0.060 and *p* = 0.638, respectively). Furthermore, the AT and CT groups significantly reduced their daily intake of mono-unsaturated fat (*p* = 0.050 and *p* = 0.039, respectively) and cholesterol (*p* = 0.041 and *p* = 0.028, respectively), whereas the NE group subjects did not (mono-unsaturated fat: *p* = 0.488 and cholesterol: *p* = 0.937). All group participants were found to have unchanged (*p* ≤ 0.05) daily intakes of polyunsaturated fat and fibre at post-test.

The AT group were found to have significant increases in the weight lifted during shoulder press (*p* = 0.007), latissimus dorsi pull-downs (*p* = 0.004), seated chest press (*p* = 0.003), low-pulley row (*p* = 0.005), crunches (*p* = 0.003), leg press (*p* = 0.002), knee extensions (*p* = 0.002) and hamstring curls (*p* = 0.004) (Table 2). Similarly, the CT group had significant increases in the weight lifted during shoulder press (*p* = 0.001), latissimus dorsi pull-downs (*p* = 0.001), seated chest press (*p* = 0.001), low pulley row (*p* = 0.001), crunches (*p* = 0.001), leg press (*p* = 0.001), knee extensions (*p* = 0.001) and hamstring curls (*p* = 0.001). The NE subjects had no significant (*p* ≤ 0.05) change in their measures of strength from baseline to post-training.

Data indicated that the VO_2max_ of the groups were homogenous at baseline (*p* = 0.113). The VO_2max_ of the NE subjects did not change significantly from baseline to post-training (*p* = 0.347). However, both the aerobic training and combination training subjects improved their VO_2max_ significantly (*p* = 0.003; *p* = 0.001; respectively).

## Discussion

The primary finding of the current investigation is that 16 weeks of moderate-intensity aerobic training performed for 45 minutes three times weekly, or a combination of aerobic and resistance training performed at a moderate intensity for a total of 45 minutes three times weekly equally lowered blood concentrations of LDL-C in healthy, previously untrained men. Similarly, several other studies,^6^-^12^ and especially that of Desprès *et al.*^36^ who utilised a comparable research design observed that aerobic training could lower LDL-C concentrations.

The effect of resistance training on lipoprotein lipid profiles is as yet not well documented, neither are the results as consistent as those for aerobic training.^18^ Some, but not all^14^,^15^,^18^-^21^ resistance training studies have reported lower LDL-C concentrations.^5^,^17^,^22^-^25^,^37^

There is no evident difference in study design (in terms of samples and exercise variables) between those studies that did not significantly alter LDL-C concentrations, implying that there are no definite variables ensuring success in lowering LDL-C concentrations. Due to the novelty of a combination of aerobic and resistance training being investigated for its effects on LDL-C and lipoprotein lipids, only the study of Le Mura *et al*.^8^ was found to contradict the present study’s findings. This may be due to the present investigation making use of moderate-intensity exercise and not high-intensity exercise as in the case of Le Mura *et al*.^8^ (> 70% of VO_2max_ and 60–70% 1-RM). This increased intensity may have led to glycogen being used as the primary fuel source instead of relying on the necessary fat metabolism utilised during low to moderate intensity.^8^

According to Durstine and Haskell,^38^ exercise-induced modification in levels of LDL-C, its subfractions and its apolipoproteins are only minor without change in adiposity of dietary fat or cholesterol intake. The majority of exercise studies that demonstrated favourable alterations in lipoprotein lipid profiles with exercise were due to a loss of body weight and primarily a loss in body fat mass or increases in VO_2max_.^17^,^18^ In this study, the NE and AT group participants demonstrated a significant decrease in body mass, whereas the CT group subjects’ body mass remained unchanged. No significant relationship was found between blood LDL-C concentrations and body mass (*r* = 0.020).

While the NE group was found to have no change in percentage body fat, both the AT and CT groups demonstrated significant decreases in percentage body fat, leading to a significant moderate relationship being found between blood LDL-C concentrations and percentage body fat (*r* = 0.350). Furthermore, the findings of this study corroborate those of Hurley *et al*.^17^ and Joseph *et al*.^18^ who suggested that most changes in LDL-C are due to an increase in VO_2max_. The changes, or lack thereof, in lipoprotein lipids may also be due to potential dietary changes.^8^,^20^ In this regard, the significant changes in the total fat intake following aerobic and combination training resulted in a significant moderate relationship being found between blood LDL-C concentrations and total fat intake (*r* = 0.321). While the CT group significantly altered their daily intake of saturated fat, the AT group remained unchanged.

A significant moderate relationship was found between the blood LDL-C concentrations and saturated fat intake (*r* = 0.478). Despite the AT and CT group participants significantly reducing their daily intake of mono-unsaturated fat and cholesterol, no significant relationships were found between changes in blood LDL-C concentrations and changes in daily intakes of mono-unsaturated fat (*r* = 0.235) and cholesterol (*r* = 0.237). As all groups were found to have unchanged daily intakes of polyunsaturated fat and fibre, no significant relationships were found between these dietary variables and blood LDL-C concentrations (*r* = 0.023 and *r* = 0.038, respectively). Both the AT and CT programmes significantly decreased LDL-C while at the same time increasing VO_2max._

The findings of the present investigation support the hypothesis that aerobic training can be used to lower blood LDL-C concentrations. However, we also found that a combination of resistance and aerobic training was effective in lowering blood LDL-C concentrations in this sample of healthy, previously untrained men. This is noteworthy, since a combination of aerobic and resistance training decreased blood concentrations of LDL-C by almost the same amount as aerobic training alone, despite the fact that the subjects who engaged in the combined training did only half as much aerobic training as the aerobiconly training group.

It is likely that by combining resistance and aerobic training, the present investigation not only resulted in an increased lipid metabolism, but also decreased protein loss and the maintenance of or increase in metabolic rate,^18^,^25^ which could have assisted in the decreased LDL-C concentrations with this mode of training. The present finding that combined aerobic and resistance training may be able to reduce the incidence of dyslipidaemia, while at the same time eliciting gains in more than one physiological system and improving several health-related components increases the importance of the use of this mode of training.
